# Hepatotoxicity and pharmacokinetics of cisplatin in combination therapy with a traditional Chinese medicine compound of Zengmian Yiliu granules in ICR mice and SKOV-3-bearing nude mice

**DOI:** 10.1186/s12906-015-0799-9

**Published:** 2015-08-18

**Authors:** Can Gong, Lin Qian, Hong Yang, Li-li Ji, Hai Wei, Wen-bin Zhou, Cong Qi, Chang-hong Wang

**Affiliations:** The Institute of Chinese Materia Medica, Shanghai University of Traditional Chinese Medicine, The Ministry of Education (MOE) Key Laboratory for Standardization of Chinese Medicines and Shanghai Key Laboratory of TCM Complex Prescription, 1200 Cailun Road, Zhangjiang Hi-Tech Park, Shanghai, 201203 People’s Republic of China; Department of Gynaecology, Shanghai Shuguang Hospital Affiliated with Shanghai University of Traditional Chinese Medicine, 528 Zhang Heng Road, Zhangjiang Hi-Tech Park, Shanghai, 201203 People’s Republic of China; Research Center for Traditional Chinese Medicine and Systems Biology, Shanghai University of Traditional Chinese Medicine, 1200 Cailun Road, Zhangjiang Hi-Tech Park, Shanghai, 201203 People’s Republic of China; School and Chemical and Environmental Engineering, Shanghai Institute of Techanology, 100 Haiquan Road, Fengxian Shanghai, 201418 People’s Republic of China

**Keywords:** Cisplatin, Hepatotoxicity, Pharmacokinetics, Zengmian Yiliu granule

## Abstract

**Background:**

Cisplatin (CDDP) is a highly effective chemotherapeutic agent used for therapy of many tumors and has been limited by its toxicity. Zengmian Yiliu granule (ZMYL), a compound preparation of traditional Chinese medicines, has been used in clinic as a complementary and alternative medicine for attenuating CDDP-induced toxicities and enhancing the tumor therapeutic effect of CDDP. The aim of the present study is to investigate hepaprotective effect of ZMYL against CDDP-induced hepatotoxicity. Further, the pharmacokinetic characteristics of CDDP in SKOV-3-bearing nude mice were observed.

**Methods:**

The ICR mice were dosed orally with ZMYL for 7 days and then CDDP was injected intraperitoneally at a dose of 45 mg/kg body weight. The serum alanine aminotransferase (ALT) and aspartate aminotransferase (AST) levels were measured to evaluate the liver function. The total glutathione (T-GSH), reduced glutathione (GSH) and glutathione S-transferase (GST) levels were determined to evaluate the oxidant damage in liver homogenates. Tissue pathological change in liver was conducted by light microscopy analysis. The pharmacokinetic and tissue distribution of free and total platinum (Pt) after dosing of CDDP alone and combination with ZMYL were determined in SKOV-3-bearing nude mice by ICP-MS.

**Results:**

Oral administration of ZMYL prior to the CDDP treatment could prevent the CDDP-induced in lifting of ALT and AST, reduction of T-GSH, R-GSH and GST, and some histopathological alterations in ICR mice. Some differences in pharmacokinetic parameters between the two groups have been observed in higher *CL* and decreased *MRT* of free platinum (Pt) in plasma and total Pt in spleen in CDDP co-administration with ZMYL group. It indicated CDDP was cleared more quickly from blood and spleen, and could reduce the accumulation and toxic possibility of CDDP in combination with ZMYL.

**Conclusions:**

ZMYL could be used as a beneficial supplement, which could attenuate CDDP-induced hepatotoxicity during CDDP chemotherapy and did not disturb the pharmacokinetics fate of CDDP significantly.

**Electronic supplementary material:**

The online version of this article (doi:10.1186/s12906-015-0799-9) contains supplementary material, which is available to authorized users.

## Background

As a first-generation platinum (Pt) antitumor agent, cisplatin (cis-diaminedichloroplatinum, CDDP) has been widely used in chemotherapy either alone or in combination with other chemotherapeutic agents or with radiotherapy for treatment of a variety of different malignancies, including testicular cancer, ovarian cancer, (non-) small cell lung cancer, head and neck cancer and bladder cancer [1].

Despite its excellent anticancer activity, the clinical use of CDDP is often limited by its undesirable side effects, such as severe hepatotoxicity and nephrotoxicity [2, 3]. CDDP is preferentially taken up and accumulated in liver and kidney cells [4], resulting in the decreased antioxidant enzymes [5–7]. The generation of free radicals is believed to be a critical mechanism involved in the development of CDDP toxicity [8]. Glutathione (GSH) is the most important intracellular endogenous thiols used as free radical scavengers to clear free radical induced by CDDP, which maintains cell integrity and participation in the cell metabolism to avoid oxidative damage [9]. Glutathione S-transferases (GSTs) are multifunctional enzymes which play a key role in cellular detoxification by conjugating to glutathione, thereby neutralizing the electrophilic sites and rendering the products more water-soluble [10]. The elevated GSH and GST play an important role in protecting CDDP-induced hepatotoxicity.

In many cases, chemotherapy or radiotherapy alone cannot achieve a satisfactory therapeutic outcome, namely to achieve complete remission of tumors and refrain from severe side effects at therapeutically effective doses [11]. So, a number of protective agents have been investigated in clinic. These modulators should not interfere with the anti-tumor activity, should not be toxic by themselves and should be pharmacologically compatible with platinum compounds [12]. Several attempts have therefore been made to further develop ancillary drug with the aim to preserve the high therapeutic activity of CDDP by reducing the undesired side-effects, such as sunitinib, which could improve chemotherapeutic efficacy and ameliorate CDDP-induced nephrotoxicity in experimental animals [13]. CDDP combined with compound recipe of TCM also achieved satisfactory results in pharmacological synergism and toxic attenuation [14].

Zengmian Yiliu granules (ZMYL), a compound preparation composed of 13 traditional Chinese medicines including Radix Astragali Mongolic, Radix Codonopsis, Radix Rehmanniae, Herba Scutellariae Barbatae, Rhizoma Atractylodis Macrocephalae and so on, has been proved by long-term clinical application and verification for more than twenty years for curative effect in ovarian cancer. Some previous pharmacodynamics studies revealed that ZMYL could regulate the immune function and improve the bone marrow suppression in tumor bearing mice destroyed by CDDP [15], inhibit neoangiogenesis of tumor vessels [16] and reverse the CDDP-resistance of resistant ovarian cancer in nude mice [17]. These results indicated that ZMYL could be as a potential complementary and alternative medicine for CDDP chemotherapy, the protective effect of ZMYL in CDDP-induced hepatotoxicity is also needed to be investigated further.

In our previous pharmacokinetics studies, ZMYL produced a potential drug-herbs interaction on pharmacokinetic parameters of CDDP calculating from the total and free Pt concentration and Pt plasma protein binding ratios in the early stages in normal rats [18]. However, the pharmacokinetics of drug may be influenced by the pathological status, such as tumor-bearing status [19], type 2 diabetes [20] and non-alcoholic fatty liver disease [21]. Heretofore, no any study about the preclinical pharmacokinetic study for drug-herbs interaction on CDDP and ZMYL has been reported in pathological status. So the pharmacokinetic characteristics of platinum originating from CDDP in SKOV-3-tumor-bearing nude mice were studied by means of elemental platinum analysis using ICP-MS to examine the rationality of co-chemotherapy between CDDP and ZMYL. In addition, the possibility of ZMYL attenuating CDDP-induced acute hepatotoxicity in ICR mice was assessed by measuring biochemical variables, conducting histological studies and investigating the concentration of total glutathione (T-GSH), reduced glutathione (R-GSH), and glutathione S-transferase (GST) in liver.

## Methods

### Drugs

CDDP was purchased as a powder injection (QiLu Company, ShanDong, China). ZMYL was provided by Shanghai Shuguang Hospital Affiliated with Shanghai University of Traditional Chinese Medicine. Platinum standard was purchased from NSI solutions incorporated (1000 ppm, NSI, USA). The internal standard solution (100 ppm, Li, Sc, Ge, Y, In, Tb, Lu, Ir, Bi.) and tuning solution (1 ppb, Ce, Co, Li, Mg, Tl, Y.) for ICP-MS analysis were purchased from Agilent Technologies (Agilent, Tokyo, Japan). Nitric acid (HNO_3_) was obtained from Merck (65 %, Suprapur® grade, Merck, Germany). All other chemicals and reagents were of analytical grade or high performance liquid chromatography (HPLC) grade. All dilutions were made with high purity deionized water (18.2 MΩ) obtained from a Milli-Q water purification system (Millipore, MA, USA).

### Animals

Six-week-old female BALB/c-nude mice, the male and female ICR mice (25 – 30 g, 5 weeks of age) were provided by the Experimental Animal Centre of Shanghai University of Traditional Chinese Medicine, China, with a certificate of SYXK (Shanghai) 2009-0069, and were fed with free access to standard chow diet and water ad libitum. All the mice were allowed 1 week to acclimatize prior to the experiments (five animals per cage), and were housed at 21 °C, with a relative humidity of 60 % under standard environmental conditions (12 h light–dark cycle). The animal studies were performed in accordance with the regulations of experimental animal administration issued by the State Committee of Science and Technology of People’s Republic of China on 14 November 1988 and approved by the Animal Ethics Committee of Shanghai University of Traditional Chinese Medicine (No. SUTCM-2011-1010, approved at 10 October 2011).

### Preparation of ZMYL

ZMYL with batch number of 20110526, a finished product, was supplied by the Department of Pharmaceutical, Shanghai Shuguang Hospital Affiliated with Shanghai University of Traditional Chinese Medicine. The preparation process of ZMYL has been described previously [18]. To prepare of ZMYL, *Astragalus membranaceus* (Fisch.) Bge. *Var. mongholicus* (Bge.) Hsiao (18 g) was extracted via circumfluence extraction with 60 % ethanol for 1.5 h, and was then filtered. This procedure was repeated thrice, and the filtrates were ultimately combined. The residue, which was left after ethanol was evaporated, was incorporated with ten other Chinese crude drugs, namely, *Codonopsis pilosula* (Franch.) Nannf (15 g), *Paeonia lactiflora* Pall. (15 g), *Rehmannia glutinosa* Libosch*.* (12 g), *Scutellaria barbata* D.Don (12 g), *Akebia quinata* (Thunb.) Decne (12 g), *Atractylodes macrocephala* Koidz (9 g), *Lycium barbarum* L*.* (9 g), *Asparagus cochinchinensis* (Lour.) Merr (9 g), *Cornu cervi degelatinatum* (9 g), and *Aucklandia lappa* Decne (6 g), and was extracted with eight volumes of water for 1.5 h and then filtered. This extraction was repeated thrice, and the filtrates were combined and decompressed to a density of 1.1–1.2. These filtrates were precipitated using 70 % ethanol, left to stand, and filtered. Afterwards, the filtrates were merged with the ethanol extract from *A. membranaceus*, decompressed to a density of 1.1–1.2, mixed with 4.1 kg of dextrin, and spray-dried. A finished product of ZMYL was obtained and used for the experiment. The contents of paeoniflorin, albiflorin, scutellarin, astragaloside IV, and wogonin in ZMYL were 1.73, 1.38, 0.10, 0.02, and 0.002 mg/g, respectively, determined by a validated UPLC/MS method.

### Attenuation of CDDP-induced acute hepatotoxicity in mice by ZMYL

#### Acute hepatotoxicity induced by cisplatin

ICR mice were randomly divided into six groups, ten mice for each: PBS-treated group (control, A), CDDP-treated group (B), CDDP plus high dose ZMYL-treated group (C), CDDP plus middle dose ZMYL-treated group (D), CDDP plus low dose ZMYL-treated group (E) and ZMYL-treated group (F).

The ICR mice were dosed orally with ZMYL in PBS for 7 days with ZMYL (4 g/kg BW) in PBS in group F, ZMYL (6 g/kg BW) in PBS in group C, ZMYL (4 g/kg BW) in PBS in group D, and ZMYL (2 g/kg BW) in group E, respectively. One hour after the ultima treatment with ZMYL, CDDP was injected intraperitoneally at a dose of 45 mg/kg body weight (which has been reported to induce hepatotoxicity and nephrotoxicity in mice without lethality [22] in groups B, C, D and E. The control group received PBS instead of ZMYL and CDDP. All mice were killed under anesthesia 16 h later of CDDP treatment. After collecting blood of each mouse from the angular vein, livers were excised immediately. The liver tissues were perfused immediately with ice-cold saline (0.9 % NaCl) in order to remove the blood for the subsequent experiments.

#### The evaluation of hepatic damage

##### Histological studies

The specimens of the livers were fixed by immersion in 10 % formalin in 0.1 M phosphate buffer (pH 7.4) for 24 h, dehydrated, embedded in paraffin wax for tissue sectioning. The tissue sections (5 μm) were underwent hematoxylin-eosin (H&E) staining for structural evaluation by light microscopy at 200 × magnification.

##### Determination of serum biochemical parameters

The blood samples were left to stand at room temperature for 1 h, and then centrifuged at 15,700 × *g* for 10 min to obtain the serum. The serum alanine aminotransferase (ALT) and aspartate aminotransferase (AST) levels were measured to evaluate the liver function. All biochemical assays were performed by spectrophotometrically using an automatic photometer (PRIME, BPC BioSed S.r.l., Italy).

##### The total glutathione, glutathione and glutathione S-transferase levels in liver

In order to evaluate the oxidant damage in liver homogenates, the total glutathione (T-GSH), reduced glutathione (GSH) and glutathione S-transferase (GST) levels in liver were assessed spectrophotometrically according to the manufacturer’s instructions, a commercially available kits from Nanjing Jiancheng Bioenginearing Institute (Nanjing, China). The results of T-GSH, R-GSH and GSSG were expressed in mmol · g^-1^ protein. Data of GST were expressed as U/mg protein. The total protein concentration was determined using an assay kit from Nanjing Jiancheng Bioenginearing Institute, and results were normalized for total protein concentration.

### Pharmacokinetic and tissue distribution studies of CDDP in SKOV-3-bearing nude mice

#### Cell culture and construction of SKOV-3-bearing nude mice model

Human ovarian cancer SKOV-3 cells were cultured in RPMI-1640 medium, containing 10 % heat-inactivated fetal calf serum, 100 U/ml penicillin and streptomycin in an incubator with a humidified atmosphere containing 5 % CO_2_ at 37 °C [16].

Female athymic nude mice were inoculated subcutaneously (s.c.) in armpit of right forelimb with fragments (2 ± 3 mm in diameter) of the human ovarian cancer SKOV-3 cells (4 × 10^6^ cells/mouse). The study was started when tumors had reached a mean size of approximately 0.065 cm^3^ (ranged from 0.044 to 0.081 cm^3^) [15, 17].

#### Pharmacokinetic study

Experiments were performed on one hundred and eight female SKOV-3-bearing nude mice that were randomly divided into two groups of fifty-four for each according to the tumor volume, CDDP group and CDDP combining with ZMYL granules group. In CDDP group, CDDP was freshly dissolved in saline solution and was intraperitoneally administrated into mice at dose of 18 mg/kg. In CDDP combining with ZMYL granules group, ZMYL was dispersed to suspension in saline solution and administrated by *gavage* at dose of 2 g/kg to mice for successively seven days. One hour after oral administration of ZMYL at the seventh day, CDDP at dose of 18 mg/kg was administration as in CDDP group. Blood (in each case approximately 0.8 ml) was sampled from the angular vein in heparinized polythene tubes at following time points of 0.25, 0.33, 0.5, 1, 2, 6, 12, 24, and 48 h after CDDP administration, under condition of the animals unrestrained and conscious. The blood samples were immediately centrifuged at 15,700 × *g* for 10 min to obtain plasma. Plasma ultra-filtrate was obtained by centrifuging the partial plasma fraction through a 10-KDa ultra-filtrate filter [23] (Millipore Corporation, Billerica, MA, USA) for 40 min (4000 × *g*, 24 °C). Both of the plasma and plasma ultra-filtrate were stored at -80 °C until analysis.

Simultaneously, liver, spleen, kidney and tumors were removed and then rinsed in ice-cold saline before gently blotting on absorbent paper. Tissues were stored at -80 °C until analysis of platinum.

#### Preparation of plasma and tissues samples

20 μl of plasma sample/or ultra-filtrate were pre-digested with 750 μl of 65 % (v/v) HNO_3_ overnight in screw-capped digestion jar (cleaned using 35 % (v/v) HNO_3_) and then digested by microwave. Digestion was conducted with power of 800 W at 110 °C for 15 min, after deflation, continued with power of 800 W at 110 °C for 5 min, and then with power of 1600 W at 160 °C for 15 min. After completing digestion, transferred fluid from digestion jar and metered volume by 5 % nitric acid to 10 ml. After vortex mixing, the aliquot was injected directly for analysis by ICP-MS.

25 mg of tissue were pre-digested with 750 μl of 65 % (v/v) HNO_3_ overnight in screw-capped digestion jar (cleaned using 35 % (v/v) HNO_3_) and then digested by microwave. Digestion was conducted with power of 1600 W at 110 °C for 15 min, after deflation, continued with power of 1600 W at 110 °C for 5 min, and then with power of 1600 W at 160 °C for 15 min. After completing digestion, transferred fluid from digestion jar and metered volume by 5 % nitric acid to 10 ml. After vortex mixing, the aliquot was injected directly for analysis by ICP-MS.

#### Determination of elemental platinum by ICP-MS

The ICP-quadrupole-MS of Agilent 7500CX (Agilent Technologies, USA) equipped with a peristaltic pump, a nebulizer, a spray chamber and a quartz torch was used in Pt analyses. Instrumentation of MARS X for microwave digestion (CEM, USA) with digestion jar of XPRESS TFM (10 ml) was used in pretreatment of plasma sample. Ultrafiltration tube of Amicon® Ultra (10 KD) (millipore, USA) was applied for getting free Pt plasma.

Full validation of ICP-MS according to the FDA guidelines was, as far as applicable for ICP-MS, performed for the assay [18]. In this study, the extraction recoveries and matrix effects for tissues (liver, spleen, kidney, and tumor) were validated.

### Data analysis

All the data were processed using the pharmacokinetics data analysis software program PK solutions 2™ (Summit Research Services, USA), and the dose of platinum was used in the calculation of these parameters. All data were presented as means with their standard deviation. The plasma concentration - time curves were plotted. The following pharmacokinetic parameters including elimination rate constant (*Ke*), elimination half-life (*T*_*1/2*_), apparent volume of distribution (*Vd*), clearance rate (*CL*) and mean residence time (*MRT*) and other parameters were calculated. Area under the plasma concentration-time curve from zero to time t (*AUC*_*0–t*_) was obtained directly from the observed concentration-time data. The area under the plasma concentration–time curve from zero to infinity (*AUC*_*0−∞*_) was calculated by means of the linear trapezoidal rule with extrapolation to infinity with terminal elimination rate constant (*Ke*).

The results are expressed as mean values ± the standard deviation (S.D). The SPSS version 15.0 software (SPSS Inc., Chicago, IL, USA) was used for statistics. The significance of differences of experimental data of the pharmacokinetic study was assessed by Student’s *t*-test and with α = 0.05 as the minimal level of significance. Differences between treatments of experimental data of the pharmacodynamics study were determined by analysing the data using one-way ANOVA repeated measures. A statistical probability of P-0.05 was considered to be as the minimal level of significance.

## Results and discussion

### ZMYL attenuates CDDP -induced hepatotoxicity in mice

#### Tissue pathological analysis

**Fig. 1:**
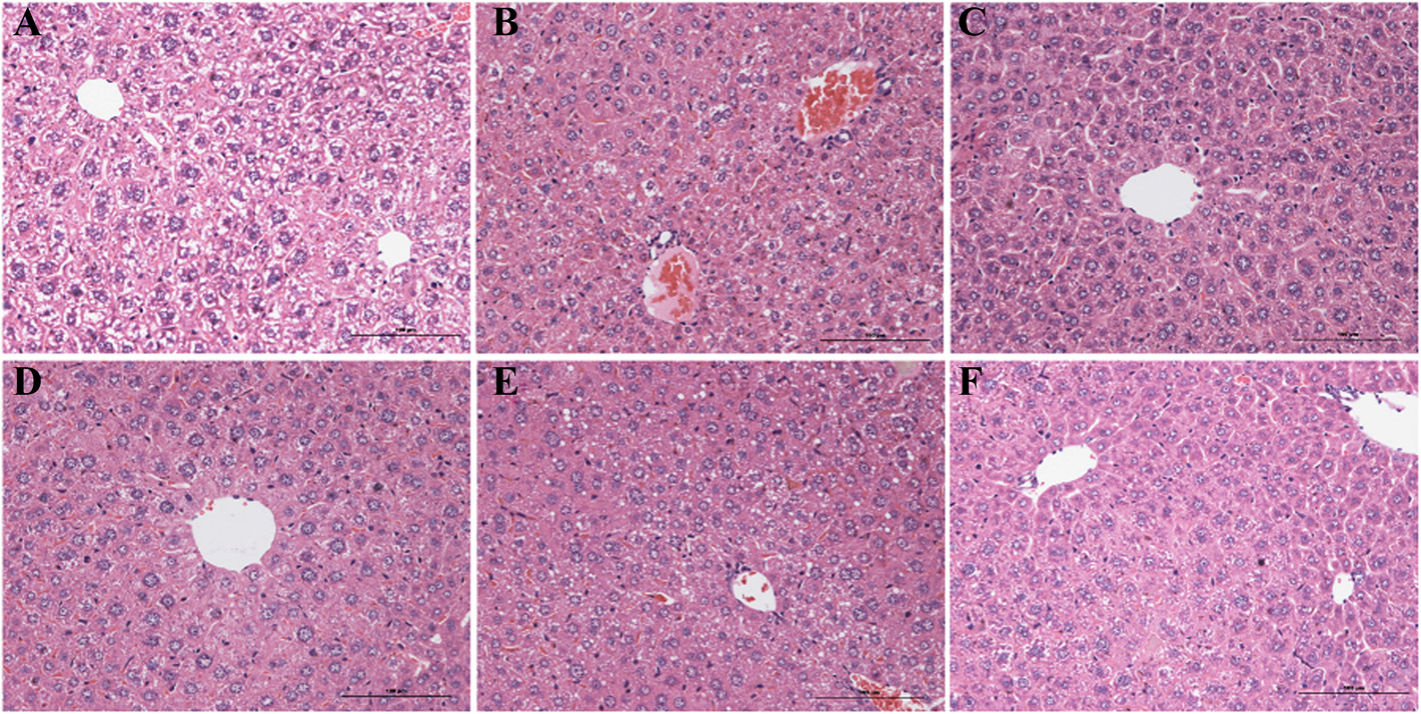
The H&E staining of protective effects of ZMYL against CDDP-induced hepatotoxicity in histological appearance of liver in ICR mice. (**a**): PBS-treated group (control), (**b**): CDDP-treated group, (**c**): CDDP plus ZMYL (6 g/kg)-treated group, (**d**): CDDP plus ZMYL (4 g/kg)-treated group, (**e**): CDDP plus ZMYL (2 g/kg)-treated group, (**f**): ZMYL (4 g/kg)-treated group. Magnification × 200

The control group showed normal histology after H&E staining (Fig. 1a). Light microscopy analysis revealed that in liver sections from mice treated with CDDP, CDDP treatment alone induced some alterations of the liver parenchyma when compared to both control and ZMYL-treated mice. CDDP induced some areas of necrotic hepatocytes, inflammatory cells infiltration and bile duct proliferation, and hepatocytes swelling. In addition, congestion was slightly more visible in CDDP group than in control group, and the structure of hepatic lobule confused (Fig. 1b). It was consistent with CDDP-induced mild pathological change in liver reported previously [24]. After treatment with ZMYL (6 g/kg BW), the liver parenchyma was quite comparable with that of the control group, except for inflammatory cells infiltration, (Fig. 1c). In liver sections from mice treated with ZMYL (4 g/kg BW), some of the hepatocytes were a little bigger, the structure of hepatic lobule is not very clear (Fig. 1d). In CDDP plus low dose ZMYL group (2 g/kg BW), congestion was found and also with unclear hepatic lobule (Fig. 1e).

#### Serum ALT and AST levels

**Fig. 2:**
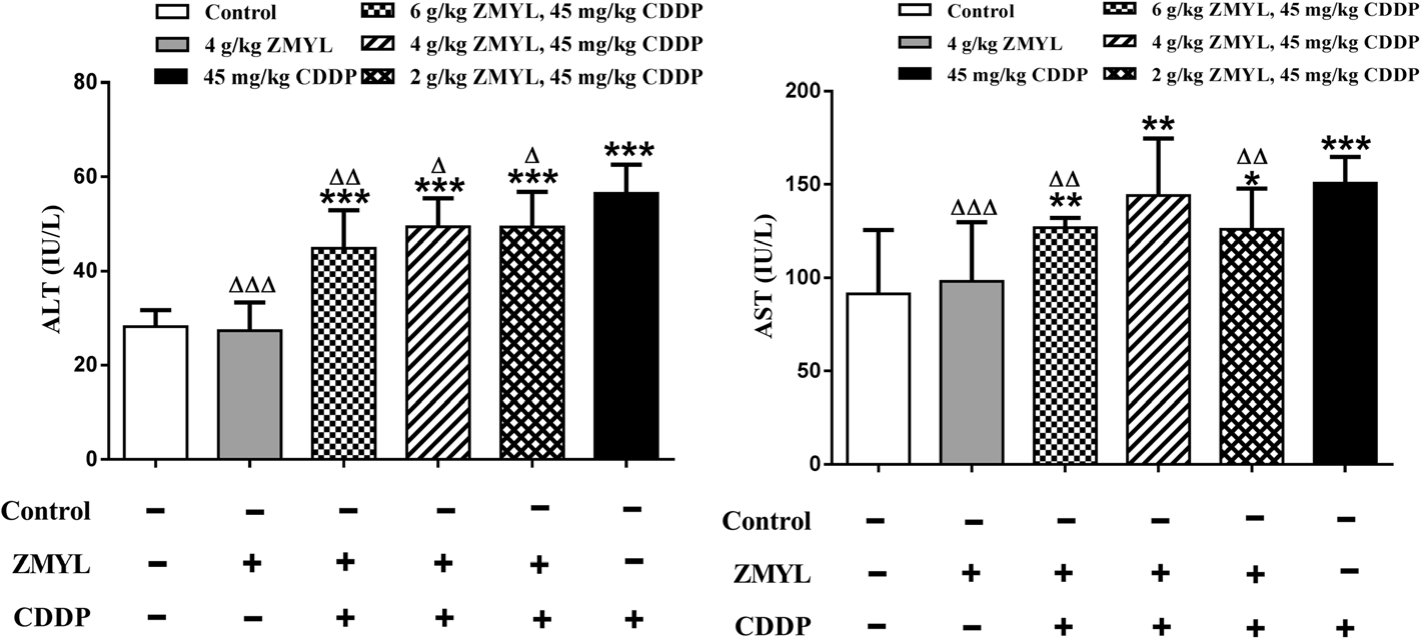
Effect of ZMYL on the serum ALT and AST levels in the CDDP -treated mice. The comparison of ALT/AST with control: *P < 0.05, **P < 0.01, ***P < 0.001; The comparison of ALT/AST with CDDP: ^**Δ**^ *P* < 0.05, ^**ΔΔ**^ *P* < 0.01, ^**ΔΔΔ**^ *P* < 0.001

These histological abnormalities were coincided with increased activity of ALT and AST. The effects of ZMYL on CDDP-mediated increases in ALT and AST in mice 7 days post-treatment were shown in Fig. 2.

In PBS-treated mice, serum ALT or AST levels were low (Fig. 2). CDDP at 45 mg/kg body weight could induce mice hepatotoxicity at 16 h after administration of CDDP, which could led to approximately two-fold enhancement in the value of ALT compared to the control (*P* < 0.001). Administration of ZMYL to mice resulted in the obvious reduction in ALT. The combination of ZMYL and CDDP treatment could decrease ALT levels about 20.55 % (*P* < 0.01), 12.60 % (*P* < 0.05) and 12.69 % (*P* < 0.05) in CDDP plus ZMYL (6 g/kg BW)-treated group, CDDP plus ZMYL (4 g/kg BW)-treated group, CDDP plus ZMYL (2 g/kg BW)-treated group, to contrast with the CDDP-alone treatment mice (Fig. 2).

In the experiments with mice treated with CDDP alone, a 1.65 fold augmentation (*P* < 0.001) was observed in AST compared to the control. In the combination of ZMYL and CDDP group, treatment with ZMYL could result in the obvious reduction in AST induced by CDDP. The combination of ZMYL and CDDP treatment could decrease AST levels about 15.82 % (*P* < 0.01) and 16.41 % (*P* < 0.01) in CDDP plus ZMYL (6 g/kg BW) -treated group and CDDP plus ZMYL (2 g/kg BW)-treated group, to contrast with the CDDP-alone treatment mice (Fig. 2). The above-mentioned liver changes were significantly attenuated by ZMYL treatment in the CDDP + ZMYL group.

#### CDDP-induced liver oxidant stress and activity of antioxidant enzymes

**Fig. 3:**
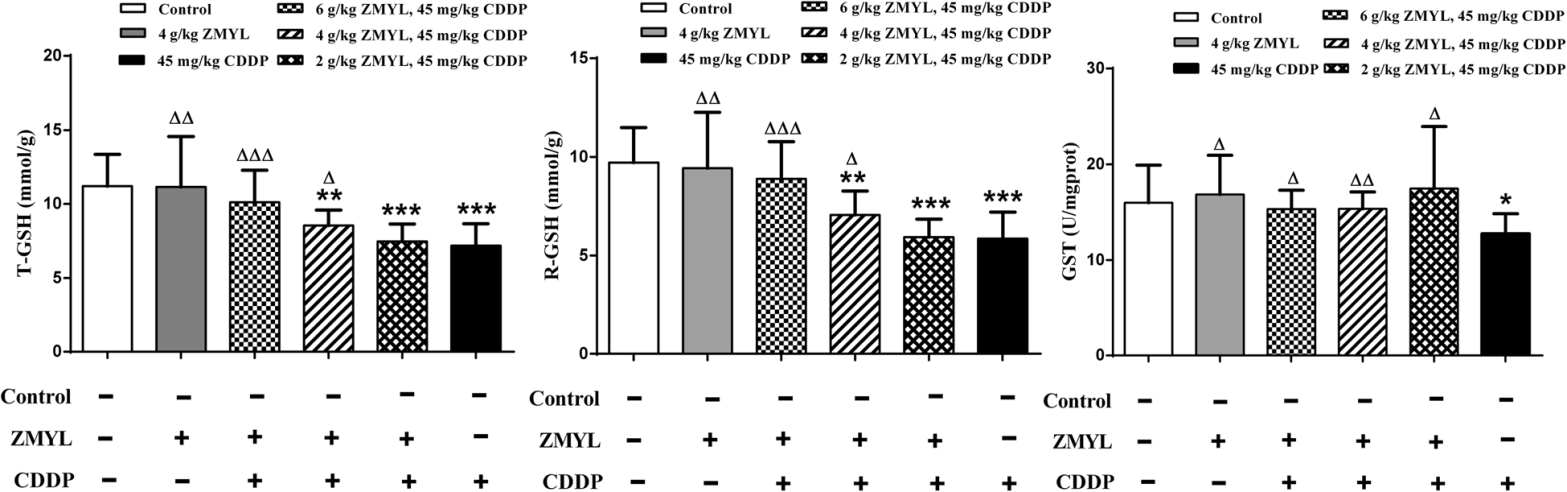
Effect of ZMYL on T-GSH / R-GSH / GST level in liver. The comparison of T-GSH / R-GSH / GST with control: *P < 0.05, **P < 0.01, ***P < 0.001; The comparison of T-GSH / R-GSH / GST with CDDP: ΔP < 0.05, ΔΔP < 0.01, ΔΔΔP < 0.001

The effect of ZMYL on CDDP-mediated changes in the levels of T-GSH, R-GSH and GST enzymatic activity in mice were showed in Fig. 3. Administration of ZMYL alone had no statistically effects on T-GSH, R-GSH levels and GST enzymatic activity as compared to the control group.

The CDDP treatment could result in a 36.07 % decrease in T-GSH at 16 h after CDDP administration compared to control (*P* < 0.001). ZMYL could induce an increase on CDDP-mediated the decrease of T-GSH in CDDP plus ZMYL (4 g/kg BW)-treated group (*P* < 0.05) and ZMYL (2 g/kg BW)-treated group (*P* < 0.001), to contrast with the CDDP-alone treatment mice. T-GSH content was decreased, but not significantly in CDDP plus ZMYL (6 g/kg BW)-treated group, compared to control. The levels of T-GSH were approximately normal levels in the mice treated with ZMYL.

Figure 3 showed the changes in R-GSH levels in the livers. Compared with the control group, the levels of R-GSH were significantly lower in group administered CDDP (*P* < 0.001). This decrease was elevated by pretreatment with ZMYL. R-GSH levels increased about 1.52 (*P* < 0.001) and 1.21 fold (*P* < 0.05) in CDDP plus high dose ZMYL-treated group and CDDP plus middle dose ZMYL-treated group, to contrast with the CDDP-alone treatment mice.

The reduced levels of GST activities were seen in the liver of CDDP-treated mice compared with the control group. ZMYL pretreatment could alleviate these CDDP-induced decreases (Fig. 3). The levels of GST were restored to approximately normal levels in the mice treated with ZMYL plus CDDP, and GST levels were elevated 24.29 % (*P* < 0.05), 20.60 % (*P* < 0.01) and 37.34 % (*P* < 0.05) in CDDP plus ZMYL (6 g/kg BW)-treated group, CDDP plus ZMYL (4 g/kg BW)-treated group, CDDP plus ZMYL (2 g/kg BW)-treated group, to contrast with the CDDP-alone treatment mice.

### Pharmacokinetics study of CDDP in SKOV-3-bearing nude mice

#### Validation of analytical methods

The calibration curve of platinum was linear within the ranges from 0.001 to 1000 ng/ml in both plasma and tissue samples (r > 0.9999). The correlation coefficient of standard curve was greater than 0.9999. The extraction recoveries at three QC levels were determined by comparing concentrations obtained from tissues (C), which were spiked with the analytes prior to extraction, with those of standard solution (B) at the same concentration. The value C/B × 100 % was considered as the extraction recoveries. The extraction recoveries for tissue from QC samples at low (0.1 ng/ml), medium (1 ng/ml) and high concentrations (100 ng/ml) were 90.63–102.21 % for liver, 96.68–103.11 % for spleen, 86.28–101.94 % for kidney, 101.70–106.90 % for tumor, respectively. The extraction recoveries for plasma were 101.62–102.83 % for plasma, 97.67–101.30 % for plasma ultra-filtrate.

The matrix effects was investigated by comparing of each analyte (at three concentration levels of QC samples) into extracts originating from tissues (B) to those of the same analyte presented in the 5 % nitric acid (A). The value B / A × 100 % was considered as the matrix effects. In terms of matrix effect, all of the ratios defined above were within acceptable limits (97.04–102.37 % for liver, 100.03–108.66 % for spleen, 103.70–113.29 % for kidney, 96.22–103.81 % for tumor, 100.57–108.75 % for plasma, 101.56–107.47 % for plasma ultra-filtrate). After dilution, there was no significant matrix effect for plasma and tissues observed.

The ICP-MS method described above has been proved to be sensitive, selective, and rapid for determination of platinum originating from CDDP in mice plasma, and tissue. The optimized method was validated to guarantee the need of the determination.

#### The CDDP plasma concentration-time profiles and pharmacokinetic parameters

**Fig. 4:**
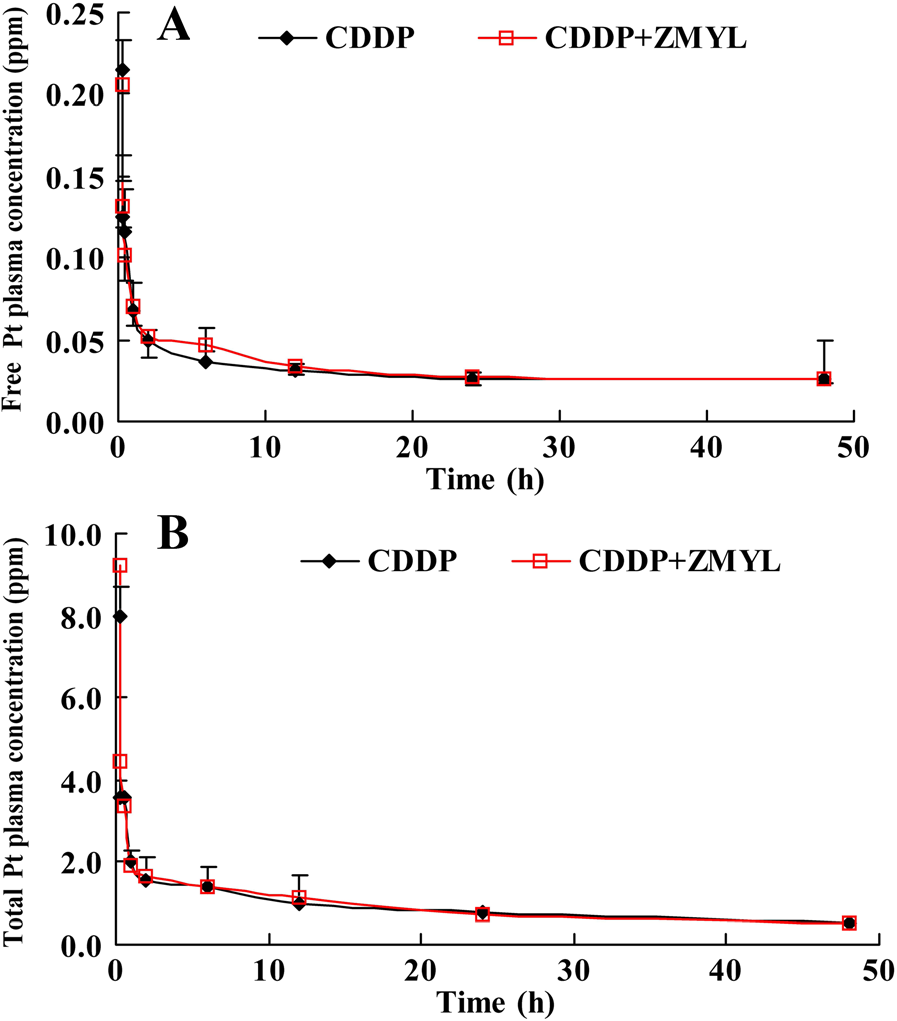
The mean plasma concentration–time profiles of unbound (**a**) and total (**b**) Pt originating from CDDP in mice following intraperitoneally administration of CDDP at dose of 18 mg/kg and co-administration with ZMYL at dose of 2 g/kg (mean ± SD, *n* = 6)

The plasma concentration-time curves of free and total Pt of CDDP after dose of 18 mg/kg and co-administration with ZMYL at dose of 2 g/kg in SKOV-3-bearing nude mice were shown in Fig. 4. After intraperitoneally administration of CDDP at dose of 18 mg/kg and co-administration with ZMYL at dose of 2 g/kg in tumor-bearing mice, a long terminal elimination phase of free Pt originating from CDDP was observed (Fig. 4a), and no significantly change was found in both CDDP alone group and CDDP co-administration with ZMYL group. The plasma concentrations of total Pt originating from CDDP exhibited decreasing rapidly within the first 2 h and then declined slowly with a long terminal phase (Fig. 4b). The peak total Pt concentrations were reached in plasma within 15 min after intraperitoneally administration for both groups, which was in agreement with several previous reports [25]. The flat terminal phase was observed, probably due to the slow redistribution of platinum from tissue back to plasma. In the absence and presence of ZMYL, ZMYL pretreatment did not significantly change the total and free Pt plasma concentration-time curves.

The plasma pharmacokinetic parameters of total and free Pt calculated from plasma of CDDP alone and combination groups were listed in Table 1. To compare the pharmacokinetic parameters of CDDP from ZMYL combination group to CDDP alone group, it was found that no significant differences were found in the parameters for total Pt between the CDDP alone and ZMYL combination-treated groups. The main pharmacokinetic parameters of free Pt between the two treatment groups demonstrated that ZMYL could significantly impact pharmacokinetic and pharmacodynamic properties of unbound CDDP. There were notable differences between the pharmacokinetic parameters in the two groups, mainly including *MRT*, *CL*, *T*_*1/2*_, and *Ke*. It was easy to find that the *MRT* reduced from 1857.4 to 409.1 h, the *CL* increased from 358.9 to 1417.6 mL · h^-1^ · kg^-1^, the *T*_*1/2*_ decreased from 1295.7 to 290.2 h, and *Ke* increased from 0.001 to 0.002 h^-1^ respectively, in ZMYL combination group. Higher *CL* of free Pt in CDDP co-administration with ZMYL group indicated which was cleared more quickly from blood and could reduce the accumulation and toxic possibility of CDDP by combination with ZMYL.

**Table 1:** Mean pharmacokinetic parameters of platinum originating from CDDP in plasma and plasma ultra-filtrate after intraperitoneally administration of CDDP at dose of 18 mg/kg and co-administration with ZMYL at dose of 2 g/kg (mean ± SD, *n* = 6)

Pharmacokinetic parameters	CDDP		CDDP + ZMYL	
	Free pt	Total pt	Free pt	Total pt
AUC_(0~48)_ (μg · h/mL)	1.5	44.6	1.6	46.6
MRT (h)	1857.4	61.6	409.1	57.3
V_d_ (L/kg)	671.018	15.2	593.758	14.883
CL (mL/h/kg)	358.891	223.575	1417.647	225.295
T_1/2e_ (h)	1295.703	47.117	290.252	45.778
Ke (1/h)	0.001	0.015	0.002	0.015

#### Tissue distribution of CDDP in SKOV-3-bearing nude mice

**Fig. 5:**
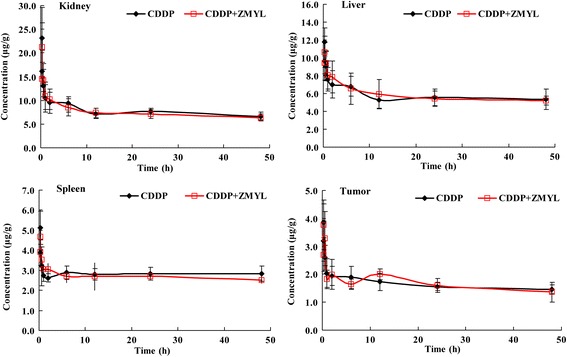
Mean concentrations of platinum in tissues at 0.25-48 h in mice of CDDP group and CDDP + ZMYL group after intraperitoneally administration of CDDP at dose of 18 mg/kg and co-administration with ZMYL at dose of 2 g/kg (mean ± SD, *n* = 6)

As shown in Fig. 5, the concentration of total Pt was determined at nine different time points including 0.25, 0.33, 0.5, 1, 2, 6, 12, 24, and 48 h after CDDP administration. The concentration of total Pt in liver, spleen, kidney, and tumor declined with the time points. The results indicated that CDDP could distribute to different tissues in a time-dependent manner. The distribution process of CDDP to tissues was very quickly, and then eliminated slowly with a long terminal phase. The highest CDDP levels were found in the kidney, then the liver, spleen, and tumor.

The main pharmacokinetic parameters of the two treatment groups calculated from the tissue concentrations from 0 – 48 h after administration of CDDP were listed in Table 2. There was no notable difference on main pharmacokinetic parameters of CDDP in liver and kidney between the CDDP alone and ZMYL combination-treated groups.

**Table 2:** Mean pharmacokinetic parameters in tissues after treatment with CDDP (18 mg/kg) alone or in combination with ZMYL (2 g/kg)

Pharmacokinetic parameters	CDDP				CDDP + ZMYL			
	Liver	Kidney	Spleen	Tumor	Liver	Kidney	Spleen	Tumor
AUC_(0~48)_ (μg · h/mL)	276.7	373.3	135.9	79.2	279.5	359.4	129.6	78.6
MRT (h)	525.7	176.9	6165.3	351.6	594.6	209.5	294.4	146.6
V_d_ (L/kg)	3074.4	2059.0	6333.5	10704.4	3164.8	2232.1	6121.4	9492.2
CL (mL/h/kg)	5.832	11.577	1.027	30.229	5.293	10.55	20.806	64.65
T_1/2e_ (h)	365.331	123.252	4272.58	245.402	414.337	146.622	203.886	101.75
Ke (1/h)	0.002	0.006	0	0.003	0.002	0.005	0.003	0.007

In spleen, the *AUC*_(0~∞)_ value of total platinum was 17522.2 μg · h · mL^-1^ for CDDP alone group while 865.1 μg · h · mL^-1^ for combination group. *MRT* of CDDP group was 6165.3 h, while that of combination group was 294.4 h. *CL* for CDDP group was 1.03 mL · h^−1^ · kg^−1^, while 20.81 mL · h^−1^ · kg^-1^ for combination group. The *T*_1/2_ value of CDDP group was 4272.58 h, while that of combination group was 203.89 h.

Table 2 showed the pharmacokinetic parameters of total platinum in tumors of CDDP alone group and CDDP combination with ZMYL group. The *MRT* of total platinum in mice were 351.6 and 146.6 h respectively, *T*_1/2e_ were 245.40 and 101.75 h respectively, *CL* were 30.23 and 64.65 mL · h^−1^ · kg^−1^, and *Ke* increased from 0.003 to 0.007 h^−1^ respectively in CDDP alone group and CDDP combination with ZMYL group.

#### The plasma protein binding of CDDP in SKOV-3-bearing nude mice

**Fig. 6:**
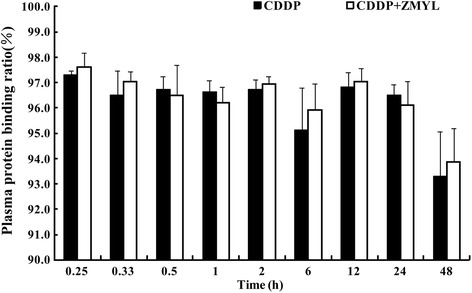
Pt plasma protein binding rate after administration of CDDP between CDDP alone group and ZMYL combination group (means ± SD, *n* = 6)

The CDDP plasma protein binding rate in both CDDP alone group and CDDP co-administration with ZMYL group for the individual sampling time point were determined (Fig. 6). The bound CDDP fractions within 48 h after administration of CDDP were stable relatively at about 93.30 % to 97.31 % for CDDP alone group, 93.87 to 97.62 % for ZMYL combination group, respectively. So that more than 90 % of CDDP was present in the plasma as the bound drug by 48 h. The results were identical with those reported in literature in that the CDDP binds rapidly to plasma proteins and after 24 h administration CDDP, more than 95 % of CDDP is protein-bound [26].

CDDP is a platinum-based chemotherapy drug used to treat various types of cancers, such as germ cell tumors, sarcomas and lymphomas. Currently, the only way to overcome acquired resistance to CDDP in cancer cells is to increase the dosage, which results in higher toxicity to normal body cells. The side effects of CDDP affect the quality of life of patients and compromise the ability of physicians to deliver adequate doses of effective chemotherapy [26]. Recent studies suggest that using traditional Chinese medicine (TCM) in combination with CDDP can enhance the effect of chemotherapeutic agents and/or lower their toxicity [27]. Therefore, a number of natural products with antioxidative activity have been evaluated against CDDP-induced toxicity to seek complete protective agents [26]. ZMYL is an herbal composition designed to supplementing Qi and nourishing Yin, clearing away the heat-evil, and expelling superficial evil in patients with ovarian cancer in combination with CDDP. ZMYL was tested for its efficacy as complementary and alternative medicine. The possibility of ZMYL both to enhance the efficacy of CDDP while reducing its side effects and to ameliorate CDDP-resistent in nude mice bearing human ovarian cancer xenografts was proved previously [15, 17].

In the present study performed in ICR mice, the single administration of a high-dose CDDP (45 mg/kg BW) over a 16 h period caused prominent liver damage, characterized by histopathological and biochemical alterations including in increases in the serum ALT and AST levels, and in damage in liver oxidant stress. Many studies have reported these alterations in the liver similar to these here [11].

Previous studies have reported that free radicals are responsible for a wide range of chemotherapy-induced side effects, including liver toxicities. CDDP can cause the generation of oxygen free radicals in the liver. These radicals can cause extensive tissue damage as they reacting with macromolecules in the liver [28]. CDDP could cause toxicity in various organs by disturbing the oxidant/antioxidant balance [26], and CDDP decreased GSH levels in liver suggesting that oxidant stress is involved in the mechanisms of CDDP-induced hepatotoxicity [11]. Similar results were reported by in rats [28], rabbits [29], and mice [30] models, respectively. In these studies, CDDP also increased ALT and AST serum activities and induced liver oxidant stress.

In general, GSH and GST play important roles in biological systems to protect against oxidative stress. GSH is a ubiquitous thiol-containing tripeptide that plays a key role in cell biology. It modulates cell responses to redox changes associated with the generation of reactive oxygen species, regulates apoptotic cell death, and mediates transmembrane transport of organic solutes. The significant reduction in GSH levels promoted by CDDP represents an alteration in the cellular redox state, suggesting that the cells could have become more sensitive to reactive oxygen species (ROS). This would lead to a reduction in effectiveness of the antioxidant enzyme defense system [28].

The decreased GSH level increases the sensitivity of organ to oxidative and chemical injury [26]. The decrease in GSH content could also be a direct factor in CDDP-induced oxidant damage to liver [11]. GST represents a large family of multifunctional proteins that are essential for disposal of exogenous toxic compounds and for adaptive antioxidant responses to ROS. This enzyme catalyzes the reaction of compounds with the sulfhydryl group of glutathione, creating products that are more water-soluble [28].

The repeated oral administration of ZMYL prior to the CDDP treatment prevented the CDDP-induced alterations in these functional indices in the combination groups in response to oxidative stress. The effect of ZMYL on CDDP-induced oxidative stress was also evaluated by determining the level of T-GSH, R-GSH and GST depletion in the liver tissues. ZMYL in combination with CDDP treatment prevented the loss of CDDP -mediated T-GSH, R-GSH and GST in the liver tissues. The effects of ZMYL on GSH in the liver may therefore be due directly to its antioxidant effects or may represent enhanced biosynthesis of GSH. The present results demonstrate that ZMYL may protect against the CDDP-induced hepatotoxicity by reducing oxidative stress in mice. However, further studies are needed to determine the exact mechanism of ZMYL on CDDP-induced liver damage. ZMYL supplementation may have a potential therapeutic role for decreasing the side effects of liver toxicity resulting from administration of cancer medicines.

It is well-known that preclinical pharmacokinetic study including plasma pharmacokinetics, tissue distribution, and protein binding study of a compound can be extrapolated to human and helps us to understand and predict its behavior in clinic [31]. There were some differences in pharmacokinetic parameters between the two groups. Higher *CL* and decreased *MRT* of free Pt in plasma and total Pt in spleen in CDDP co-administration with ZMYL group indicated which was cleared more quickly from blood and spleen, and could reduce the accumulation and toxic possibility of CDDP by combination with ZMYL. Higher *CL* of tumor in CDDP co-administration with ZMYL group was also observed. This seemed to be related to that ZMYL could influence the pharmacokinetic properties of CDDP in tissues (Table 2). The main pharmacokinetic parameters of free Pt between the two treatment groups demonstrated that ZMYL was helpful in reducing CDDP-induced toxicity.

In our previous pharmacokinetics studies in normal rats [18], ZMYL could produce a potential pharmacokinetics drug-herbs interaction on pharmacokinetic parameters of CDDP and plasma protein binding ratios in the early dosing stages (at 0.17, 0.33, 0.5, 1 h after dosing of CDDP). Drugs are used to treat diseases and only patients are the ultimate consumers of drugs, there were some differences in pharmacokinetics process between pathological state and normal condition. In recent years, more and more research shows that the pharmacokinetic parameter of drugs can be affected by the disease states, such as the PK properties of everolimus differed between normal and tumor-bearing rodents [19]. The pharmacokinetics of berberine could be altered in irritable bowel syndrome rats, a pathological condition [32]. The pharmacokinetic profiles of lansoprazole were different between gastric ulcer and normal rabbits, including increased *AUC*_(0~∞)_, *MRT*, *T*_*1/2*_ and decreased *CL* in gastric ulcer rabbits [33]. The pharmacokinetic parameters of scutellarin were also significantly different between normal and model rats [34].

The differences in pharmacokinetics process of CDDP between pathological state and normal condition were also found between SK-OV-3-bearing nude mice model and normal rats in our study. In the present pharmacokinetics study of CDDP in SK-OV-3-bearing nude mice model, there were notable differences between the pharmacokinetic parameters for free Pt in the two groups, mainly including decreased *MRT*, *T*_*1/2*_, *Ke*, and increased *CL* respectively, in ZMYL combination group. But statistical difference (*P* < 0.05) was found in increased *Vd* and *CL* in normal rats [18]. No evident changes were found in the parameters for total Pt between the CDDP and combination-treated groups in SK-OV-3-bearing nude mice model. But the parameters from the total Pt concentration in normal rats were changed as decreased *Ke* and *CL*, extended *MRT*, and *T*_*1/2*_, respectively, in co-administration with ZMYL group (*P* < 0.05) [18]. The Pt plasma protein binding ratios in the early stages (at 0.17, 0.33, 0.5, 1 h after dosing of CDDP) were boosted significantly by combination with ZMYL in normal rats [18], but no difference was discovered in SK-OV-3-bearing nude mice model. It was proved that the pharmacokinetics process of CDDP would be affected by pathological state. It is possible that drug metabolic enzymes, transporters, cell membrane permeability and the change of microbe group could be affected by physiological and pathological changes, which enable the pharmacokinetics of drugs in the body to be altered, including the process of absorption, distribution, metabolism and excretion. In addition, ZMYL could enhance the anti-tumor efficacy of CDDP in tumor-bearing mice (see Additional file 1: Figure S1). The liver index was also decreased significantly in combination group (*P* < 0.05) and ZMYL group (*P* < 0.01) in comparison to the CDDP group (Additional file 1: Figure S2). It is implied that the protective effect of ZMYL against CDDP-induced toxicity might be come true through other pharmacodynamics mechanism, rather than through affecting the pharmacokinetic process of CDDP.

## Conclusions

In conclusion, this research investigated the pharmacokinetic and hepatotoxicity characteristics of free Pt originating from CDDP with and without ZMYL in SKOV-3-bearing mice and ICR mice. The present results would contribute to the further research about drug- herbs interaction between CDDP and ZMYL.

## Additional file

Additional file 1: ZMYL enhances the anti-tumor efficacy of CDDP in tumor-bearing mice. Figure 1 photographs of solid tumor (A), tumor weight and relative tumor volume of solid tumor (B) taken from mice treated with saline (Model), ZMYL, CDDP + ZMYL and CDDP. Data show the mean ± SD (means ± SD, *n*=9). ***p* < 0.01, **p* < 0.05: compared with the model group. Figure S2. The organ index (liver, spleen, and kidney) in SK-OV-3-bearing nude mice model of four groups (Model, CDDP, ZMYL and CDDP+ZMYL) was displayed (A). Compare the concentration of total Pt in liver, spleen, kidney in SK-OV-3-bearing nude mice model between CDDP alone group and ZMYL combination group (B). Data show the Mean ± SD ( means ± SD, *n*=9). ***p* < 0.01, **p* < 0.05 compared with model group,^##^ *p* < 0.01, ^#^ *p* < 0.05: compared with CDDP group. (DOC 2488 kb)

## References

[1] Gelderblom H, Loos WJ, Verweij J, van der Burg MEL, de Jonge MJA, Brouwer E, Nooter K, Stoter G, Sparreboom A (2002). Modulation of cisplatin pharmacodynamics by Cremophor EL: experimental and clinical studies. Eur J Cancer.

[2] Winston JA, Safirstein R (1985). Reduced renal blood flow in early cisplatin-induced acute renal failure in the rat. Am J Physiol.

[3] Chirino YI, Hernández-Pando R, Pedraza-Chaverrí J (2004). Peroxynitrite decomposition catalyst ameliorates renal damage and protein nitration in cisplatin-induced nephrotoxicity in rats. BMC Pharmacol.

[4] Stewart DJ, Benjamin RS, Luna M, Feun L, Caprioli R, Seifert W, Loo TL (1982). Human tissue distribution of platinum after cis-diamminedichloroplatinum. Cancer Chemother Pharmacol.

[5] Kim YK, Jung JS, Lee SH, Kim YW (1997). Effects of antioxidants and Ca^2+^ in cisplatin-induced cell injury in rabbit renal cortical slices. Toxicol Appl Pharmcol.

[6] Somani SM, Husain K, Whitworth C, Trammell GL, Malafa M, Rybak LP (2000). Dose-dependent protection by lipoic acid against cisplatin-induced nephrotoxicity in rats: antioxidant defense system. Pharmacol Toxicol.

[7] Mora LO, Antunes LM, Francescato HD, Bianchi ML (2003). The effects of oral glutamine on cisplatin-induced nephrotoxicity in rats. Pharmacol Res.

[8] Giridharan VV, Thandavarayan RA, Bhilwade HN, Ko KM, Watanabe K, Konishi T (2012). Schisandrin B, attenuates cisplatin-induced oxidative stress, genotoxicity and neurotoxicity through modulating NF-κB pathway in mice. Free Radical Res.

[9] Sahu BD, Rentam KK, Putcha UK, Kuncha M, Vegi GM, Sistla R (2011). Carnosic acid attenuates renal injury in an experimental model of rat cisplatin-induced nephrotoxicity. Food Chem Toxicol.

[10] Rodrigues MA, Rodrigues JL, Martins NM, Barbosa F, Curti C, Santos NA, Santos AC (2010). Carvedilol protects against the renal mitochondrial toxicity induced by cisplatin in rats. Mitochondrion.

[11] Park HR, Ju EJ, Jo SK, Jung U, Kim SH, Yee ST (2009). Enhanced antitumor efficacy of cisplatin in combination with HemoHIM in tumor-bearing mice. BMC Cancer.

[12] Boven E, Verschraagen M, Hulscher TM, Erkelens CA, Hausheer FH, Pinedo HM, van der Vijgh WJ (2002). BNP7787, a novel protector against platinum-related toxicities, does not affect the efficacy of cisplatin or carboplatin in human tumour xenografts. Eur J Cancer.

[13] Suddek GM (2011). Sunitinib improves chemotherapeutic efficacy and ameliorates cisplatin-induced nephrotoxicity in experimental animals. Cancer Chem Pharmacol.

[14] Dwivedi C, Agrawal P, Natarajan K, Sharma H (2005). Antioxidant and protective effects of Amrit Nectar tablets on adriamycin-and cisplatin-induced toxicities. J Altern Complem Med.

[15] Zhang QH, Hu XX, Qi C, Li JX (2003). Experimental study on regulating effects of Zengmian Yiliu granule on immunosuppression and myelosuppression following large-dose chemotherapy in ovarian carcinoma. Shanghai J Trad Chinese Med.

[16] Hu XX, Zhang QH, Qi C, Li JX (2012). Anti-angiogenic effects of Zengmian Yiliu granule on ovarian carcinoma xenograft. Chinese J Integr Trad Western Med.

[17] Li JX, Zhang QH, Hu XX, Qi C (2012). Effects of Zengmian Yiliu decoction on expression of resistance related genes HIF-1, Glut1, MDR1, P-gp in nude mice with cisplatin-resistant ovarian cancer. Shanghai J Trad Chinese Med.

[18] Zhang QH, Gong C, Yang H, Wei H, Zhou WB, Qi C, Wang CH (2015). Pharmacokinetics of cisplatin in the absence or presence of ZengmianYiliu granules (a traditional Chinese medicine compound) in rats determined via ICP-MS: An investigation on drug–herbs interactions. Pharmaceut Biol.

[19] O’Reilly T, McSheehy PM, Kawai R, Kretz O, McMahon L, Brueggen J, Bruelisauer A, Gschwind HP, Allegrini PR, Lane HA (2010). Comparative pharmacokinetics of RAD001 (everolimus) in normal and tumor-bearing rodents. Cancer Chemother Pharmacol.

[20] Deng YX, Shi QZ, Chen B, Zhang XJ, Liu SZ, Qiu XM (2012). Comparative pharmacokinetics of baicalin in normal and the type 2 diabetic rats after oral administration of the Radix scutellariae extract. Fitoterapia.

[21] Yang J, Lv F, Chen XQ, Cui WX, Chen LH, Wen XD, Wang Q (2013). Pharmacokinetic study of major bioactive components in rats after oral administration of extract of Ilex hainanensis by high-performance liquid chromatography/electrospray ionization mass spectrometry. J Pharmaceut Biomed Anal.

[22] Liu J, Liu Y, Habeebu SS, Klaassen CD (1998). Metallothionein (MT)-null mice are sensitive to cisplatin-induced hepatotoxicity. Toxicol Appl Pharmcol.

[23] Johnsson A, Björk H, Schütz A, Skärby T (1997). Sample handling for determination of free platinum in blood after cisplatin exposure. Cancer Chemother Pharmacol.

[24] Lu Y, Cederbaum AI (2006). Cisplatin-induced hepatotoxicity is enhanced by elevated expression of cytochrome P450 2E1. Toxicol Science.

[25] Johnsson A, Olsson C, Nygren O, Nilsson M, Seiving B, Cavallin-Stahl E (1995). Pharmacokinetics and tissue distribution of cisplatin in nude mice: platinum levels and cisplatin-DNA adducts. Cancer Chemother Pharmacol.

[26] Alderden RA, Hall MD, Hambley TW (2006). The discovery and development of cisplatin. J Chem Educat.

[27] Lee CK, Park KK, Hwang JK, Lee SK, Chung WY (2008). The extract of Prunus persica Flesh (PPFE) attenuates chemotherapy-induced hepatotoxicity in mice. Phytother Res.

[28] Gaona-Gaona L, Molina-Jijón E, Tapia E, Zazueta C, Hernández-Pando R, Calderón-Oliver M, Zarco-Márquez G, Pinzón E, Pedraza-Chaverri J (2011). Protective effect of sulforaphane pretreatment against cisplatin-induced liver and mitochondrial oxidant damage in rats. Toxicology.

[29] Cayır K, Karadeniz A, Simşek N, Yıldırım S, Karakuş E, Kara A, Akkoyun HT, Sengül E (2011). Pomegranate seed extract attenuates chemotherapy-Induced acute nephrotoxicity and hepatotoxicity in rats. J Med Food.

[30] Kart A, Cigremis Y, Karaman M, Ozen H (2010). Caffeic acid phenethyl ester (CAPE) ameliorates cisplatin-induced hepatotoxicity in rabbit. Exper Toxicol Pathol.

[31] Liao YJ, Lu XQ, Lu CW (2008). Selection of agents for prevention of cisplatin-induced hepatotoxicity. Pharmacol Res.

[32] Zhao D, Zhang Y, Xu C, Dong C, Lin H, Zhang L, Li C, Ren S, Wang X, Yang S, Han D, Chen X (2012). Pharmacokinetics, tissue distribution, and plasma protein binding study of platinum originating from dicycloplatin, a novel antitumor supramolecule, in rats and dogs by ICP-MS. Biol Trace Elem Res.

[33] Gong Z, Chen Y, Zhang R, Wang Y, Guo Y, Yang Q, Zhang H, Dong Y, Weng X, Gao S, Zhu X (2014). Pharmacokinetic comparison of berberine in rat plasma after oral administration of berberine hydrochloride in normal and post inflammation irritable bowel syndrome rats. Inter J Mol Sci.

[34] Han Q, Pan GL, Zhang YW (2011). Pharmacokinetics study of lansoprazole between gastric ulcer and normal rabbits. Pharmaceut Clinic Res.

