# Supplementary material for: Hepatotoxicity and pharmacokinetics of cisplatin in combination therapy with a traditional Chinese medicine compound of Zengmian Yiliu granules in ICR mice and SKOV-3-bearing nude mice
Source: BMC Complement Altern Med. 2015 Aug 18;15:283. doi: 10.1186/s12906-015-0799-9 (PMC4538754; doi:10.1186/s12906-015-0799-9)
Supplement: Additional file 1: ZMYL enhances the anti-tumor efficacy of CDDP in tumor-bearing mice. — Figure 1 photographs of solid tumor (A), tumor weight and relative tumor volume of solid tumor (B) taken from mice treated with saline (Model), ZMYL, CDDP + ZMYL and CDDP. Data show the mean ± SD (means ± SD, n=9). **p < 0.01, *p < 0.05: compared with the model group. Figure S2. The organ index (liver, spleen, and kidney) in SK-OV-3-bearing nude mice model of four groups (Model, CDDP, ZMYL and CDDP+ZMYL) was displayed (A). Compare the concentration of total Pt in liver, spleen, kidney in SK-OV-3-bearing nude mice model between CDDP alone group and ZMYL combination group (B). Data show the Mean ± SD ( means ± SD, n=9). **p < 0.01, *p < 0.05 compared with model group,## p < 0.01, # p < 0.05: compared with CDDP group. (DOC 2488 kb) [file 12906_2015_799_MOESM1_ESM.doc]

**Additional file 1**

**Hepatotoxicity and Pharmacokinetics of Cisplatin in Combination Therapy with a Traditional Chinese Medicine Compound of Zengmian Yiliu Granules in ICR Mice and SKOV-3-bearing Nude Mice**

Can Gong ^a,d,1^, Lin Qian ^b,1^, Hong Yang ^b^, Li-li Ji ^a^, Hai Wei ^c^, Wen-Bin Zhou ^c^, Cong Qi ^b*^, Chang-hong Wang ^a*^

^a^ The Institute of Traditional Chinese Medicine, Shanghai University of Traditional Chinese Medicine, The Ministry of Education (MOE) Key Laboratory for Standardization of Chinese Medicines and Shanghai Key Laboratory of TCM Complex Prescription, 1200 Cailun Road, Zhangjiang Hi-Tech Park, Shanghai 201203, PRC

^b^ Department of Gynaecology, Shanghai Shuguang Hospital Affiliated with Shanghai University of Traditional Chinese Medicine, 528 Zhang Heng Road, Zhangjiang Hi-Tech Park, Shanghai 201203, PRC

^c^ Research Center for Traditional Chinese Medicine and Systems Biology, Shanghai University of Traditional Chinese Medicine, 1200 Cailun Road, Zhangjiang Hi-Tech Park, Shanghai 201203, PRC

^d^ School and Chemical and Environmental Engineering, Shanghai Institute of Techanology, 100 Haiquan Road, Fengxian, Shanghai 201418 , PRC

1 These authors contributed equally to this work.

*Correspondence to: Professor Chang-hong Wang and Cong Qi, The Institute of Traditional Chinese Medicine, Shanghai University of Traditional Chinese Medicine and Department of Gynaecology, Shanghai Shuguang Hospital Affiliated with Shanghai University of Traditional Chinese Medicine, 201203 Shanghai China

Tel: 086-021-51322511, Fax: 086-021-51322519, E-mail: [wchcxm@hotmail.com](mailto:wchcxm@hotmail.com) (C.H. Wang); [qicongxzq@yahoo.com.cn](mailto:qicongxzq@yahoo.com.cn) (C. Qi)

**ZMYL enhances the anti-tumor efficacy of CDDP in tumor-bearing mice**

Female SK-OV-3-bearing nude mice were divided randomly into four groups (Control, CDDP, ZMYL and CDDP+ZMYL), and each group consisted of nine mice. In CDDP group, CDDP was injected intraperitoneally at 3 mg/kg body weight (B.W.) one time for three successive days. In ZMYL group, experimental group was administered with ZMYL by *gavage* at a final concentration of 2 g/kg (B.W.) every day. In CDDP combining with ZMYL granules group, administration of CDDP and ZMYL was as same as in CDDP group and ZMYL group. While the control group received only physiological saline.

On the 21th day after initial injection CDDP, all mice of each group were sacrificed, respectively. To evaluate tumor weight and tumor size, tumor weight was measured, the tumor size was calculated as follows: tumor size= πab^2^/6, where a and b is the larger and smaller diameters, respectively. For the evaluation of response, relative tumor volumes were used which were calculated using the formula V_T_/V_0_, in which V_T_ is the volume on any given day and V_0_ is the volume at the start of treatment. The mean relative volume of treated tumors and that of control tumors on day 21 of the experiment were used to calculate the difference between the groups. For the index of liver, spleen, kidney (organ/bodyweight ratio) in tumor-bearing mice model, the weights of liver, spleen, and kidney were weighted, and body weight of each mouse was recorded.


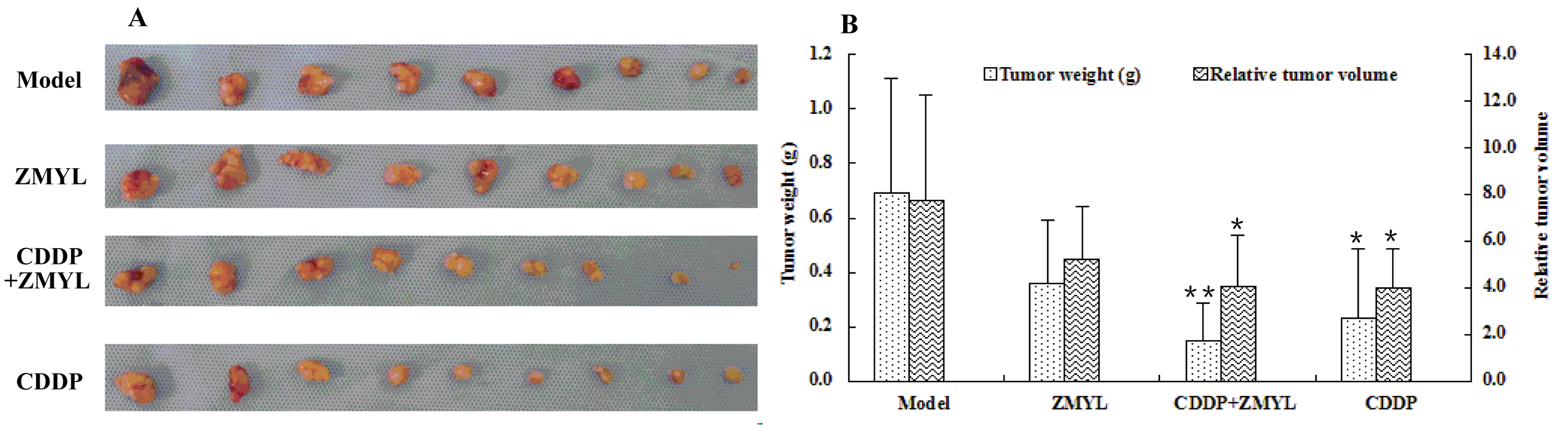


Fig. S1. Photographs of solid tumor (A), tumor weight and relative tumor volume of solid tumor (B) taken from mice treated with saline (Model), ZMYL, CDDP+ZMYL and CDDP. Data show the mean ± SD ( means ± SD, n＝9). **p < 0.01, *p < 0.05: compared with the model group.

The photographs of solid tumor in four groups (Model, ZMYL, CDDP+ZMYL and CDDP) are shown in Fig. S1A.

To assess the effect of ZMYL on tumor growth inhibition in CDDP treated SKOV-3-bearing mice, we used a tumor-bearing mice model. As shown in Fig. S1B, at 21 days after administration of CDDP (3 mg/kg), the tumor weight of the model group with saline treatment were 0.6931 ± 0.42 g. However, in the CDDP-injected group, tumor weight (0.2302 ± 0.26 g) was reduced significantly in comparison to the model group (*P* < 0.05). ZMYL supplementation with CDDP resulted in a further reduction in the tumor weight (0.1503 ± 0.14 g) (*P* < 0.01). Only ZMYL supplementation without CDDP showed no significant reduction in tumor weight (0.3587 ± 0.23 g) when compared with the model group. This may suggest that ZMYL itself did not show a significant reduction effect of tumor growth.

From the calculated relative tumor volumes on the 21th day, ZMYL combination with CDDP (4.06 ± 2.18) was clearly effective at reducing the relative tumor volume when compared with model tumor growth (*P* < 0.05). In the CDDP group, the relative tumor volume (3.97 ± 1.69) was significantly reduced (*P* < 0.05). No significant difference was present in the inhibition of tumor growth when mice were treated with ZMYL only (5.21 ± 2.30), no statistically differences were observed between the CDDP-injected group and ZMYL supplementation with CDDP group (Fig. S1B).


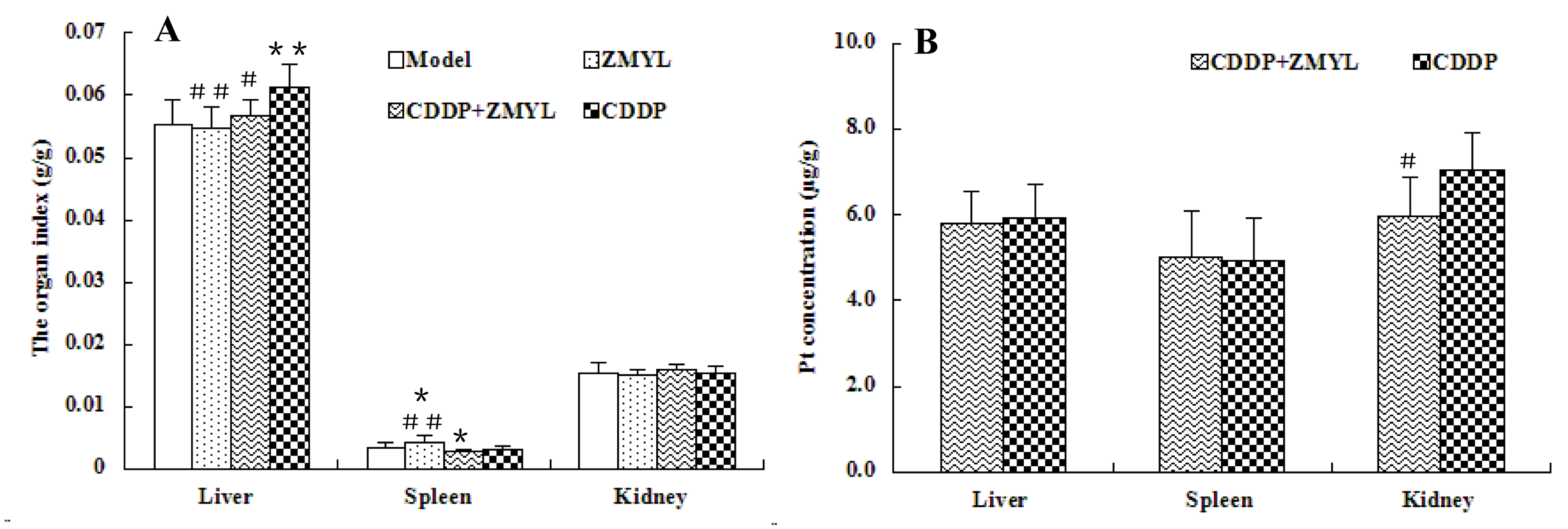


Fig. S2. The organ index (liver, spleen, and kidney) in SK-OV-3-bearing nude mice model of four groups (Model, CDDP, ZMYL and CDDP+ZMYL) was displayed (A). Compare the concentration of total Pt in liver, spleen, kidney in SK-OV-3-bearing nude mice model between CDDP alone group and ZMYL combination group (B). Data show the Mean ± SD ( means ± SD, n＝9). **p < 0.01, *p < 0.05 compared with model group，^##^p < 0.01, ^#^p < 0.05: compared with CDDP group.

In the CDDP group, the liver index (liver/body weight ratio) (0.0613 ± 0.0036 g) was increased significantly in comparison to the model group (0.0552 ± 0.0041 g) (P < 0.01), and the liver index (0.0566 ± 0.0028 g) was also decreased significantly in combination group (*P* < 0.05) and ZMYL group (0.0547 ± 0.0028 g) (*P* < 0.01) in comparison to the CDDP group. The spleen index (spleen /body weight ratio) of the ZMYL group (0.0043 ± 0.0011 g) was raised remarkably in comparison to the model group (0.0034 ± 0.0007 g) (P<0.05), ZMYL combination with CDDP group (0.0027 ± 0.0004 g) was diminished notably in comparison to the model group (0.0034 ± 0.0007 g) (*P* < 0.05), the spleen index of the ZMYL group (0.0043 ± 0.0011 g) also increased remarkably in comparison to the CDDP group (0.0030 ± 0.0006 g) (*P* < 0.01), but there was no statistically significant differences between the CDDP-injected group and the model group. There was no remarkably difference between the kidney indexes (kidney /body weight ratio) of the four groups (Fig. S2A).

CDDP and ZMYL combination group induced a significant reduction in the concentration of total Pt determined by ICP-MS in kidney, compared with CDDP given alone mice (*P* < 0.05). No statistically difference was observed for the concentration of total Pt in liver and spleen. In liver, the concentration of total Pt of CDDP group was 5.91 ± 0.74 µg/g, while that of combination group was 5.79 ± 0.80 µg/g. In spleen, the concentration of total Pt for CDDP group was 4.93 ± 0.90 µg/g, while 5.01 ± 0.86 µg/g for combination group (Fig. S2B).
